# Retinal ischemia due to different stages of atherosclerosis - insights from a retrospective study on central retinal artery occlusion

**DOI:** 10.1186/s42466-025-00413-z

**Published:** 2025-07-22

**Authors:** Felix Schlachetzki, Ina Feistenauer, Michael Ertl, Mustafa Kilic, Fabian Aden, David Pollinger, Horst Helbig, Christina Wendl, Karin Pfister, Lars Krenkel, Maria Andreea Gamulescu, Ralf Andreas Linker, Sibylle Wilfling

**Affiliations:** 1 Department of Neurology, TEMPiS Telestroke Center, University of Regensburg, medbo Bezirksklinikum, and University Hospital Regensburg, Regensburg, Germany; 2 Department of Neurology and Neurorehabilitation, University Hospital, Günzburg, Germany; 3 Department of Ophthalmology, University Hospital Regensburg, Regensburg, Germany; 4 Center for Neuroradiology, University Hospital Regensburg and medbo Bezirksklinikum, Regensburg, Germany; 5 Department of Vascular and Endovascular Surgery, University Hospital Regensburg, Regensburg, Germany; 6 Department of Biofluidmechanics, Technical University of Applied Sciences (OTH) Regensburg, Regensburg, Germany; 7 Center for Human Genetics Regensburg, Further Str. 10a, 93059 Regensburg, Germany

**Keywords:** Retinal ischemia, Spot sign, Orbital color coded sonography, Atherosclerosis, Cholesterol emboli, Central retinal artery occlusion

## Abstract

**Background:**

Ischemic stroke (IS) and retinal ischemia (IR) share similar vascular risk factors, but differ in their risk for subsequent or recurrent stroke and therapeutic options. This study characterizes the cardiovascular risk profiles and magnitude of atherosclerosis of the carotid artery of patients with central retinal artery occlusion (CRAO) in relation to the presence of the retrobulbar “spot sign” on orbital color-coded sonography (OCCS).

**Methods:**

We performed a retrospective analysis on the detailed cardiovascular risk factors and neuroimaging data in patients with IR presenting between 2009 and 2023. Based on OCCS findings, CRAO were further divided into hyperechoic (“spot sign positive”, ssCRAO) or hypoechoic CRAO (heCRAO). Statistical analyses were performed with Mann-Whitney-U and χ [2] testing. P-values were considered significant if < 0.05.

**Results:**

Overall, 112 patients were identified (heCRAO: *n* = 32; ssCRAO: *n* = 80). ssCRAO patients were significantly older (median 74 years vs. 66.5 years, Mann-Whitney-U: p-value < 0.001). Overall, 15/103 (14.6%) patients had concurrent acute ischemic stroke– 9 in the ipsilateral internal carotid territory, 2 in other territories and 4 disseminated. Further significant differences were found regarding the echogenicity of atherosclerosis (AS) in the two subgroups with (mainly) echorich AS being more common in the ssCRAO group (p-value < 0.001, *n* = 108) and the distribution of high-grade vs. low-grade stenoses of the ipsi- and contralateral carotid artery (p-value < 0.05, *n* = 99). 20 out of 112 patients had atrial fibrillation (aFib) with 17 of these being on ongoing oral anticoagulation.

**Conclusion:**

According to this study, atherosclerosis may be one of the most important risk factors for IR while a specific embolic source could not be demonstrated (i.e. acute plaque rupture). By contrast, current oral anticoagulation for aFib in CRAO patients was high, thus only an incidental finding and may be an incidental finding due to its prevalence in the elderly. Furthermore, we were able to distinguish two subgroups of IR that differ in risk factors and most likely also in etiology, therapy and prognosis. The study underlines the importance of OCCS to detect “spot signs” in IR with indications for both, acute thrombolysis and secondary prevention.

**Supplementary Information:**

The online version contains supplementary material available at 10.1186/s42466-025-00413-z.

## Background

Retinal ischemia (IR) from non-arteritic central retinal artery occlusion (CRAO) still lacks effective treatment and prognosis remains poor [1, 2]. Yet, the retina and brain share the same arterial supply and the vascular risk factors for ischemic stroke (IS) and IR are similar⁠ [3]. Nevertheless, the progress seen in IS therapy, mainly intravenous thrombolysis (IVT) and mechanical thrombectomy, has not been achieved in IR [4]. Current treatment options for IR due to CRAO are limited to “conservative” procedures [5–7]. The potential advantage of systemic thrombolysis is currently being investigated in controlled studies while local intra-arterial therapies have been abandoned due to severe complications, lack of efficacy and late presentation of patients [1, 4, 6, 8–15].

Still, the specific etiopathology and embolic source is not well defined, which is critical for secondary prevention as well as for the prognosis after recanalizing therapies in the acute setting. A promising diagnostic tool is orbital color-coded sonography (OCCS) which can identify the presence or absence of the retrobulbar “spot sign” as a marker for CRAO. A positive “spot sign” may suggest either calcified or cholesterol emboli in CRAO and branch retinal artery occlusion, and these dominantly cholesterol emboli are “Hollenhorst plaques” [16, 17]. Frequently found upon fundoscopy, they are thought to originate from atherosclerotic plaques. In fact, a pathohistological work-up of carotid endarterectomy specimen of “symptomatic” high-grade internal carotid artery (ICA) stenoses from the Oxford Plaque and Athero-Express-Study revealed significantly more features of plaque vulnerability such as large lipid core, inflammation and overall plaque instability as well as less fibrous content and calcification in patients with IS to patients with IR [18]. Thus, organ size and emboli composition may also be responsible for differences between stroke and retinal ischemia that render a direct transfer of successful therapeutic strategies from IS to IR problematic. Also retinal arteries including the central retinal artery are essentially endarteries similar to the perforating arteries of the pons and basal ganglia. This implies higher susceptibility to ischemia, difficulties for penetration and distribution of thrombolytic agents and consequently shorter therapeutic windows and the possibility of plain hypoperfusion infarction. All these factors may need to be considered for future studies.

In summary, due to differences in emboli size and composition, different responses to current medical, endovascular and surgical intervention for acute treatment and secondary prevention treatment, IR differs considerably from IS and thus, the Trial of Org 10,172 in Acute Stroke Treatment (TOAST) criteria might not be transferred directly from IS to IR [19]. The aim of this study was to identify specific cardiovascular risk factors, extracranial carotid atherosclerosis (AS) and possible causes for the emboli leading to IR in two distinct subgroups based on OCCS and fundoscopy: spot-sign positive CRAO (ssCRAO) and spot-sign negative (= hypoechogenic) CRAO (heCRAO).

## Methods

This longitudinal, retrospective study was approved by the Ethical Review Board of the University of Regensburg (reference: 19-1542-104). Discharge letters were screened for patients who suffered CRAO. Patients with arteritic CRAO and amaurosis fugax as well as patients with branch retinal artery occlusion were excluded.

The following data were extracted from the medical record: age, sex, antihypertensive medication, diabetes mellitus, hypercholesterinemia, history of smoking, peripheral artery disease, presence of persistent or intermittent atrial fibrillation (AF), cardiac abnormalities including aortic and mitral valve pathologies and patent foramen ovale, previous stenting of coronary arteries and history of myocardial infarction, anti-thrombotic and anti-platelet medication as well as oral anticoagulation at presentation. Furthermore, the patients were evaluated for peripheral emboli and further vascular events.

Any available neuroimaging by cerebral computed tomography (cCT) and cerebral magnetic resonance imaging (cMRI) was re-evaluated for the presence of acute and previous cerebral ischemia, including the vascular territory and signs of small vessel disease according to Fazekas [20].

In addition, atherosclerosis was analyzed in detail using the ultrasound image data base as IR patients were routinely scanned as part of the diagnostic work up in the stroke unit (ClinWinData, Erlangen, Germany). Neurosonographic investigations were performed according to stroke unit standard including the central retinal artery in case of CRAO by technicians and neurological residents under supervision of the attending stroke neurologists experienced in neurovascular ultrasound. The echogenicity of atherosclerotic carotid plaques (internal and common carotid arteries) was re-classified and confirmed by a DEGUM certified neurosonologist (FS) according to the Mannheim criteria using the ultrasound image archive. Any ICA stenoses were classified according to the North American Symptomatic Carotid Endarterectomy Trial (NASCET) criteria including purely B-mode graduation in < 40% stenosis as flow acceleration is lacking [21, 22]. Further analyzed parameters included the stenosis and diameter of the common carotid artery and the diameters of both vertebral arteries.

**Fig. 1:**
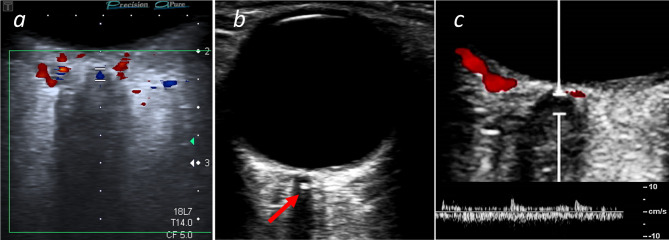
hypoechoic CRAO/heCRAO without spot sign (**figure a**) and spot sign positive CRAO/ssCRAO (red arrow, **figure b**) can be differentiated by OCCS. A positive spot sign predicts poor prognosis as well as poor response to intravenous thrombolysis and excludes an arteritic CRAO while proving an embolic cause. **Figure c** shows the corresponding flow profile with slow resistance flow profile in the central retinal artery (lower)

All patients were routinely examined with fundoscopy in the ophthalmology department and by OCCS in the neurology department as part of the standard work-up according to previously published criteria and safety guidelines [23]. In brief, the distal part of the optic nerve was identified and scanned for the characteristic “spot sign” within 3 mm from the lamina cribrosa. CRAO was additionally confirmed by absence of arterial flow in the distal optic nerve (Fig. 1).

Statistical comparison was performed with Mann-Whitney-U (two group comparisons) and Chi-squared tests (χ [2]comparison of contingency tables).

## Results

**Fig. 2:**
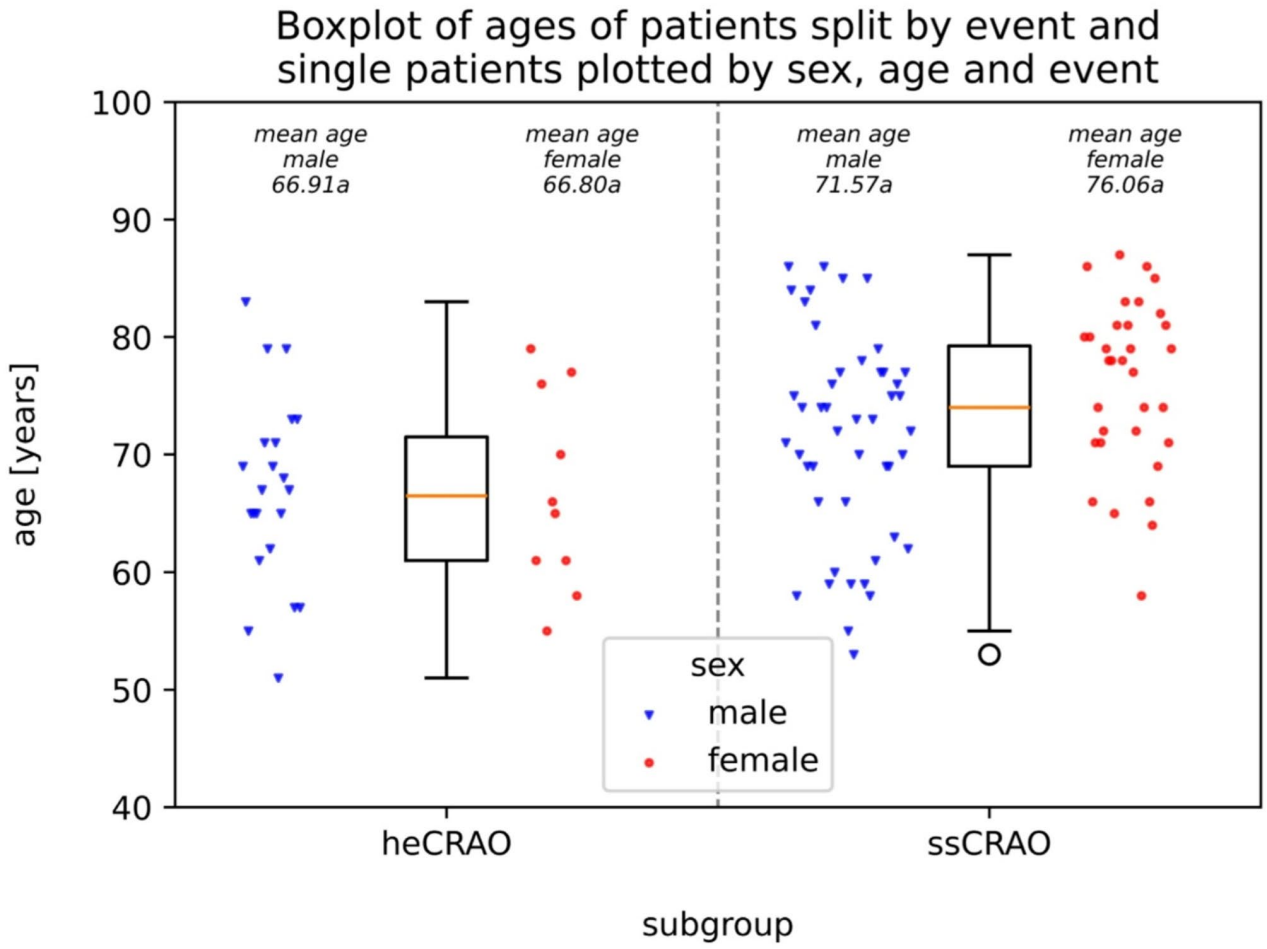
This figure shows boxplots of the ages of the patients (regardless of their sex) grouped by ocular event. The dots show every single patient for each group with blue triangles representing male and red circles representing female patients. Overall, there was a significant difference between the two subgroups regarding the ages and females showed a significantly different age distribution in the whole cohort compared to males

Overall, 112 patients treated between 2009 and 2023 were identified. The majority of patients (71%, 80 of 112 patients) had spot-sign CRAO, the heCRAO represented the smallest subgroup with 32 patients (29%). There was a significant difference in age distribution between males and females overall (mean age females = 74yrs., *n* = 43 vs. mean age males 70yrs., *n* = 69; p-value < 0.02, Mann-Whitney-U testing). Females were not significantly older on average in the ssCRAO subgroup (76yrs., *n* = 33 vs. 72 yrs., *n* = 47), but not in the heCRAO subgroup (67yrs., *n* = 10 vs. 67yrs., *n* = 22). With respect to the occurrence of ssCRAO versus heCRAO, patients with ssCRAO were significantly older (median 74yrs. vs. 66.5yrs., Mann-Whitney-U: p-value < 0.00129). Male patients were slightly overrepresented in the whole collective (69/112 patients = 61.6%) as well as in both subgroups (47/80 patients = 58.8% in ssCRAO, 22/32 patients = 68.8% in heCRAO). Boxplots of the ages split by event and single patients classified by sex, age and ocular event are shown in Fig. 2.

Overall, CT and MRI analysis in 15/103 (= 14.6%) patients showed concurrent acute ischemic events at the time of CRAO, 9 of which located in the ipsilateral middle or anterior cerebral arteries (MCA and ACA), 2 in other vascular cerebral territories and 4 disseminated above the ipsilateral ICA territory. Information on anticoagulation and antiplatelet drugs was available for 14 of 15 patients, and only 4 patients were without any. Overall, 75 of 95 patients were without old cerebral infarcts, and in 20 patients with old ischemic lesions (= 21.1%), 10 were located in the ipsilateral ACA/MCA territory, 5 in other territories and 5 disseminated.

**Fig. 3:**
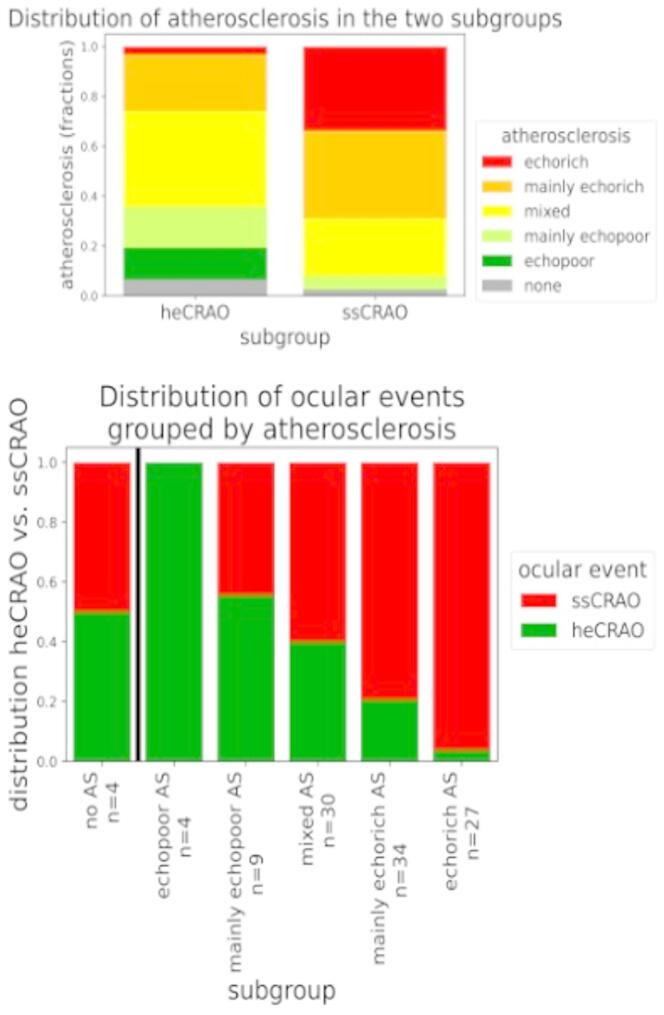
Detailed analysis of distribution of atherosclerosis and ocular events, grouped by ocular event (upper) and by atherosclerosis (lower). AS = atherosclerosis

Information on the echogenicity of atherosclerosis was available for 108 patients from the patients records based (and stroke unit standards) with confirmation by a DEGUM certified neurosonographeur (FS). χ2 revealed significant differences between the two subgroups (p-value < 0.001). In our cohort, (mainly) echorich AS is overrepresented in the ssCRAO group whereas mixed or (mainly) echopoor AS is dominant in the heCRAO group. The distribution of AS and ocular event is shown in Fig. 3, one time grouped by the type of AS and the other time grouped by ocular event.

Occurrence of atrial fibrillation, either permanent or intermittend and a major cause of severe cerebral stroke, did not differ statistically significant between the subgroups but revealed a non-significant trend towards less AF in heCRAO compared to the other two subgroups (2 out of 32 patients in heCRAO vs. 18 out of 80 patients in ssCRAO and BRAO, χ [2]: p-value ≈ 0.08). Out of the 20 patients with AF, information on medical treatment was available for 19 patients, 17 of them were treated with oral anticoagulants (with or without antiplatelet drugs) and two patients received antiplatelet drugs only. Out of the patients with AF, information about concurrent IS was available for 19/20 patients. Two of them suffered acute IS within the ipsilateral MCA/ACA territory despite oral anticoagulation. No acute events were found in other territories in all patients with AF.

Considering old ischemic events, this information was available for 18 patients with atrial fibrillation. The majority (10/18 = 55,6%) had no old cerebral infarcts while 4 patients had old events in the same ACA/MCA territory and 4 in other territories.

**Fig. 4:**
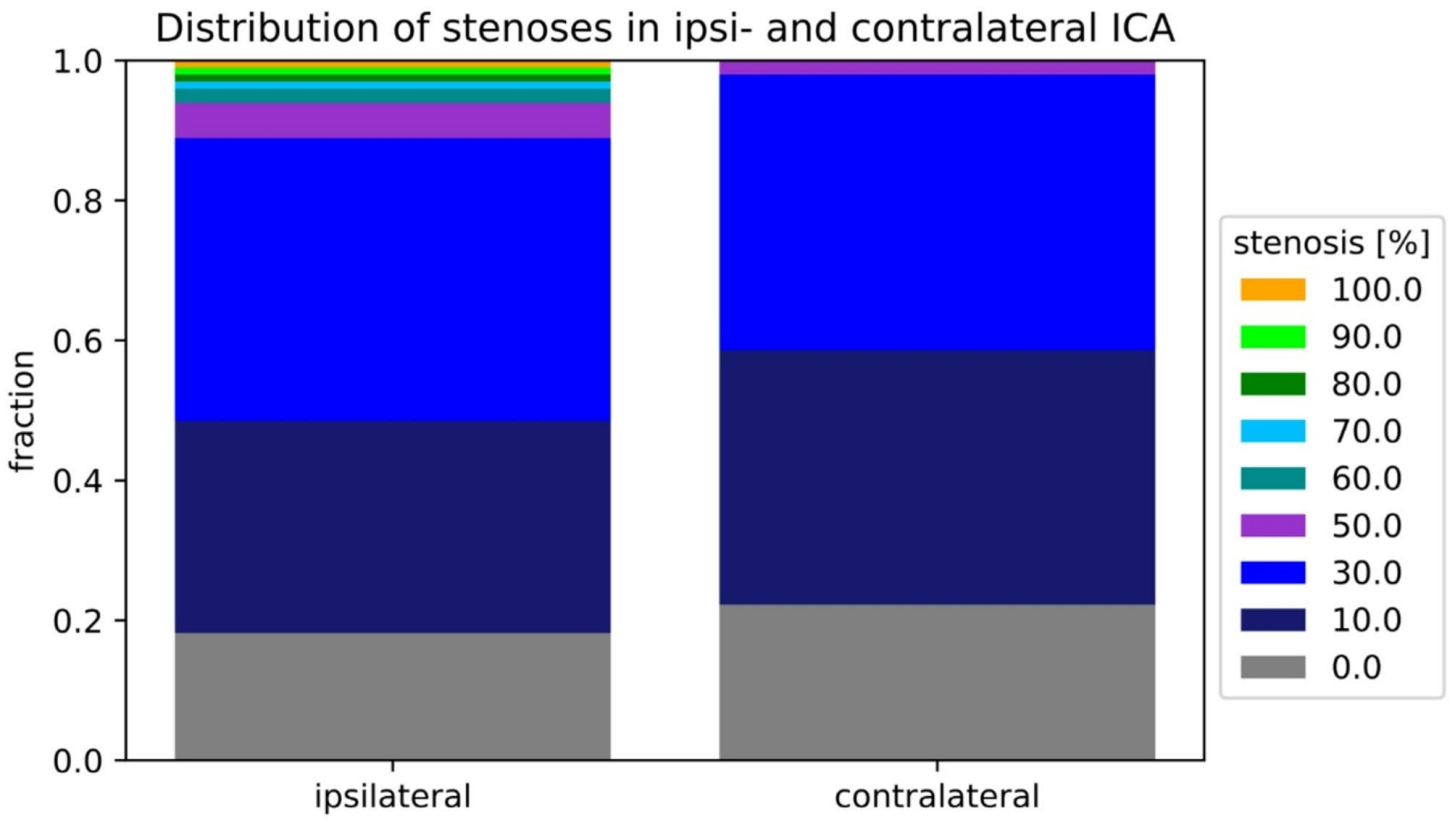
Comparison of the distribution of ICA stenoses ipsi- and contralateral. Significant differences are present when comparing high-grade (at least 50%) vs.no or low-grade stenoses in both groups. Overall, the vast majority of patients had stenoses of less than 50%

High grade ICA stenosis has a higher risk for cerebral infarcts, and information on both ICA was avalaible for 99 patients. The vast majority of patients had no or low grade ICA stenosis (defined as 50% according to NASCET, and TOAST) with ipsilateral ICA stenosis < 50% present in 88 of 99 patients (88%). Thus, an ICA stenosis larger than > 50% NASCET was observed in 11 patients and only 2 of 99 patients had an > 50% stenosis in the contralateral ICA. We observed signicant differences between hight- and low-grade stenosis as shown in Fig. 4 (p-value in chi square on contingency table < 0.22 with ≥ 50% stenosis according to NASCET).

In this retrospective study we also analyzed the presumed etiology of IR by the treating physician during the hospital stay in 105/112 cases by analyzing the discharge letter, mainly according to the TOAST criteria. 55 cases were classified as cryptogenic, 12 were attributed to AF and 34 were classified as large-artery atherosclerosis. 4 patients had other determined causes for their IR (floating structure on aortic valve, ruptured aortic plaque, calcification of bicuspid aortic valve, thrombus from mitral valve). No significant differences for a variety of variables except mean age were found between the two subgroups heCRAO and ssCRAO, see Table 1.

**Table 1:** Detailed analysis of variables grouped by ocular event. Total refers to the number of patients in the group for which information on this variable was available. CCA: common carotid artery, ICA: internal carotid artery, OAC: oral anticoagulation. * significant p-value < 0.00029 (Mann-Whitney-U testing)

	Hypoechogenic (echo poor) CRAO Patients with available information, *n* = 32		Spot-sign (echo-rich) CRAO Patients with available information, *n* = 80	
age: mean ± std	66.88 ± 7.97*	32	73.42 ± 8.43*	80
**male sex**	68.75% (22)	32	58.75% (47)	80
**echogenicity atherosclerosis**:		total: 31		total: 77
no AS	6.5% (2)		2,6% (2)	
echopoor	12.9% (4)		0% (0)	
mainly echopoor	16.1% (5)		5,2% (4)	
mixed	38.8% (12)		23,4% (18)	
mainly echorich	22.6% (7)		35,1% (27)	
echorich	3.2% (1)		33,8% (26)	
**stenosis of ipsilateral ICA**: mean(%) ± std	23.75 ± 24.06	32	22.99 ± 17.70	77
**stenosis of ipsilateral ICA**:		**total: 32**		**total: 77**
none	7		12	
10–40%	19		58	
50%	3		3	
60%	0		3	
70%	1		0	
80%	1		0	
90%	1		0	
100%	0		1	
**stenosis of ICA contralateral**: mean(%) ± std	13.45 ± 13.70	29	17.71 ± 12.76	70
**hypercholesterinemia**		total: 32		total: 79
no	15,7% (5)		21,5% (17)	
yes	50% (16)		24,1% (19)	
treated	34,4% (11)		54,4% (43)	
**hypertension**		total: 32		total: 79
no	15,7% (5)		11,4% (9)	
yes	37,5% (12)		21,5% (17)	
treated	46,9% (15)		67,1% (53)	
**diabetes mellitus**	16,7% (5)	30	26,9% (21)	78
**nicotin**		total: 21	63,5% (33)	total: 52
no	38,1% (8)		28,8% (15)	
yes	42,9% (9)		7,7% (4)	
former usage	19,0% (4)			
**peripheral artery disease**	0% (0)	31	10.1% (8)	79
**acute cerebral ischemia**		total: 31		total: 72
same territory	19,4% (6)		4,2% (3)	
other territory	0% (0)		2,8% (2)	
different territories	3,2% (1)		4,2% (3)	
none	77,4% (24)		88,9% (64)	
**OAC or antiplatelet drugs (AD)**		total: 32		total: 72
none	59,4% (19)		34,7% (25)	
AD	31,3% (10)		37,5% (27)	
OAC	3,1% (1)		23,6% (17)	
both	6,3% (2)		41,7% (3)	
**atrial fibrillation**	6.3% (2)	32	22.5% (18)	80
**patent foramen ovale**	0% (0)	31	2,5% (2)	80
**presumed etiology at discharge**		total: 32		total: 80
cryptogenic	59,4% (19)		45% (36)	
atrial fibrillation	3,1% (1)		13,8% (11)	
atherosclerotic	37,5% (12)		27,5% (22)	
other	0% (0)		5% (4)	
not defined	0% (0)		8,8% (7)	

## Discussion

In this study we differentiated CRAOs based on the pre- or absence of the hyperechoic “spot sign” in OCCS with the aim to define the most likely embolic source in order to optimize prevention and perhaps therapy [24–26]. Atherosclerosis appears to account for the majority of emboli to the retina and central retinal artery although a “specific source” such as plaque rupture could not be demonstrated. The presence of AF and the degree of carotid stenosis may be only indicative for the presence of cerebrovascular risk factors in the elderly population.

AF without anticoagulation is a strong risk factor for IS and therefore also discussed as possible etiology in IR [25]but is more common in brain than in ocular events probably due to larger emboli size [26]. Most of the patients in our cohort with AF were already on oral anticoagulation and the majority had co-existing atherosclerosis casting doubt on the causality of AF for IR. In clinical practice CRAO in these patients is discussed as failure or undertreatment of OAC leading either to change in OAC regimen (i.e. factor X inhibition to direct thrombin inhibition) or addition of antiplatelet drugs, often with increased risk of bleeding complications. However, a current observational study is currently ongoing whether or not AF is an independent risk factor for IR and on the preventive value of anticoagulation [27]. However, if the current data holds true patients with AF and IR may rather benefit from an intensification of blood pressure control and cholesterol lowering, amongst others, aiming to stabilize atherosclerosis.

**Fig. 5:**
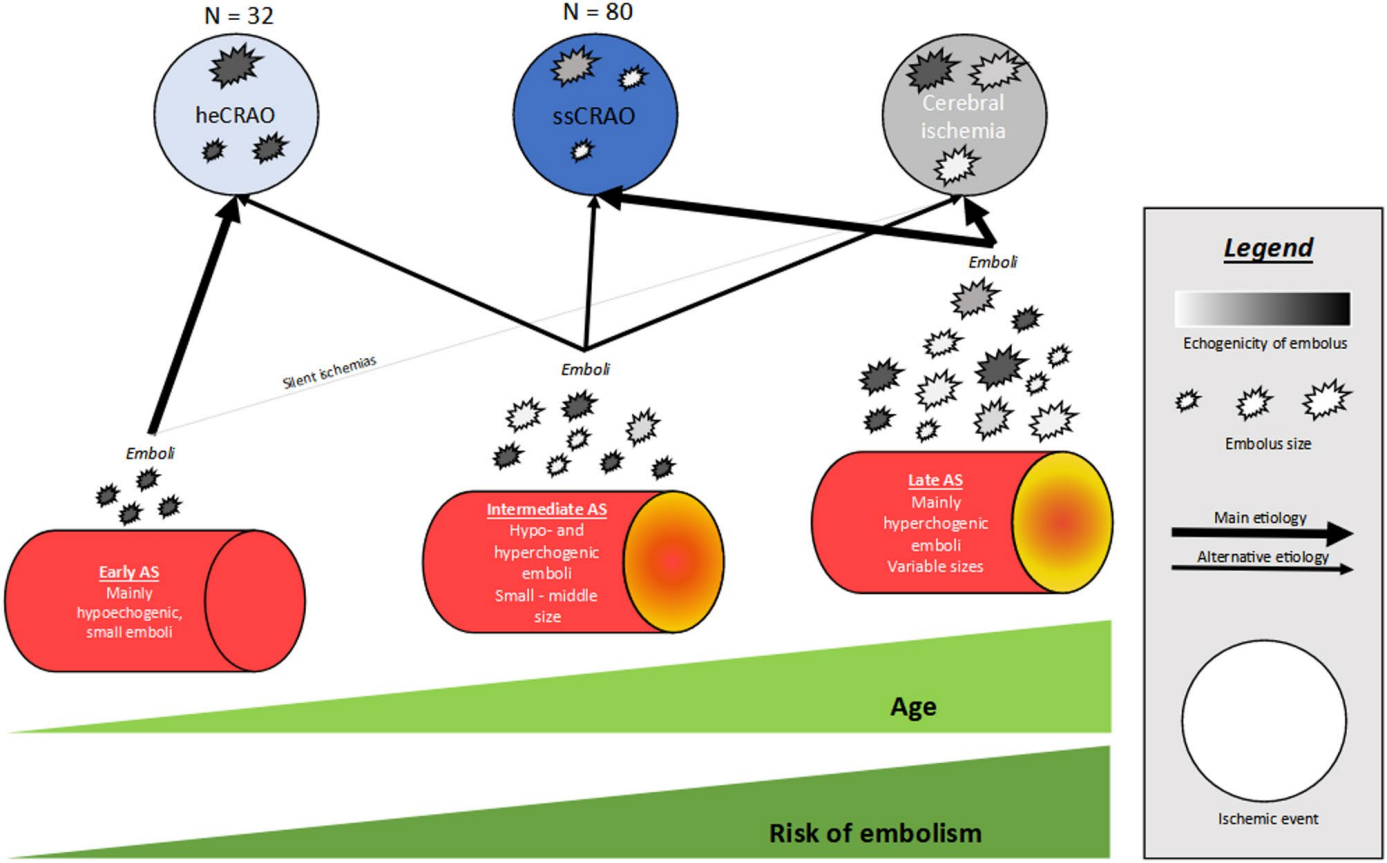
Hypothesis on the etiology of different ocular events. Atherosclerosis seems to be the main risk factor even in early stages of AS

The differentiation between ssCRAO and heCRAO exhibited differences in the age distributions and the correlating atherosclerotic risk profile. Adding data to the current discussion on atherosclerosis and sex differences, we found affected female patients to be older than affected males in the overall cohort and patients with ssCRAO being older than patients with heCRAO [28]. Based on our current and previous data, we propose the (cholesterol) emboli syndrome in atherosclerosis as pathophysiological hypothesis for a large number of CRAO, and ssCRAO can persist over years and even after IVT [29, 30]. The type of atherosclerosis seems to correlate with the clinical phenotype (ssCRAO– echorich atherosclerosis vs. heCRAO– echopoor atherosclerosis) and echorich, advanced atherosclerosis with clinically relevant stenosis is not a necessary requirement for embolic IR due to atherosclerosis. Figure 5 depicts our hypothesis on the etiology of those two ocular events. Small, echopoor emboli entering the brain might not cause (clinically relevant) ischemia given the collateral cortical network opposed to endarteries in the retina, and may be more amendable to endogenous endothelium-initiated plasminogen activator.

Given the age distribution and high rate of atherosclerosis we did not find an equivalent number of patients with embolism due to large-artery atherosclerosis as defined by the TOAST criteria which defines large-artery atherosclerosis as stenosis of brain supplying arteries higher than 50% [31]. In IS, the fraction of patients with large-artery atherosclerosis ranges from 15 to 70% [32–35]. Mead et al. speculate on pathogenetic differences in IR versus IS and found that severe ipsilateral carotid disease was less common in IS than in IR whereas atrial fibrillation was more common in ischemic stroke than in retinal ischemia [26]. This would be in-line with our cohort, although patients had significantly more high-grade stenoses in the ipsilateral ICA compared to the contralateral side, but the vast majority of patients had stenoses of less than 50%. We may hypothesized that stenoses less than 50% in the ipsilateral ICA might cause CRAO (by embolism), and could be considered “vulnerable” as recently reviewed in the context of embolic stroke of undetermined source (ESUS) [36].

In studies on symptomatic ICA stenosis, however, both transient and permanent ocular ischemia qualified as “symptomatic” but may present different types of emboli. Platelet-fibrin emboli that arise from plaque rupture and exposure of the thrombogenic subendothelial matrix have higher potential for endogenous thrombolysis with recanalization while cholesterol containing emboli or calcified plaque components are more likely to result in permanent occlusion or hypoperfusion [30]. Despite similar vascular risk profiles, differences between IS and IR are also apparent in studies on symptomatic ICA stenosis. In these patients, the risk for subsequent IS after IR lies around 50% compared to that of another IS after IS [37–39]. In the North American Symptomatic Carotid Endarterectomy Trial (NASCET) the ipsilateral stroke risk in patients after transient and permanent IR significantly resembles that of asymptomatic high grade internal carotid artery stenosis. Both types of IR are in the medical treatment arm of NASCET, casting doubt on surgical carotid endarterectomy in these patients regarding the benefit-risk ratio with comparably low risk reduction [37–39]. However, it must be noted that transient ocular symptoms such as amaurosis fugax dominate the population with respect to IR.

The percentage of concurrent acute ischemic stroke in our study population ranges within numbers given by other groups (Kim et al.: age-dependent 8–30% [33], Lee et al.: 12,1% [40], Hoyer et al.: 32,5% [41], Fallico et al.: 30% in CRAO [42]). While other studies used MRI, we mainly relied on clinical symptoms and cCT thus potentially underestimating the real number of concurrent stroke. Furthermore, we observed more, especially older cerebral infarction within same vessel territory of the affected eye, adding to the theory of atherosclerotic embolism being a more frequent cause for IR than atrial fibrillation. Laczynski et al. showed that the risk of subsequent stroke in patients with CRAO was lower than previously reported and comparable to that of all at-risk adults [43].

Given the current insufficient therapeutic options and often exceeded time frame for recanalization/ thrombolytic therapies in IR, primary prevention for IR is of highest importance. While a large body of evidence on overlapping risk factors exist in these patients, the embolic source and secondary stroke prevention is performed according to the TOAST criteria excluding (of course) microangiopathy. Also, recognition of cholesterol emboli and other atherosclerotic plaque components in acute cerebrovascular disease opposed to platelet-fibrin thrombi may have important prognostic implications for thrombolytic therapies in cerebral and retinal stroke and secondary prevention. However, presence of ssCROA should not hinder IV thrombolysis and i.e. the REVISION trial should add prognostic markers for IVT given the substudy on the presence or absence of ssCRAO [14]. It remains to be seen, whether young patients with lower frequency of ssCRAO benefit more from thrombolysis and whether or not the risk of other complications from IVT (i.e. bleeding complication, angioedema) outweigh the risk of rapid IVT without OCCS to rule out ssCRAO.

Carotid endarterectomy has a strong secondary prophylactic effect in symptomatic high-grade ICA stenosis. However, in the current study the number of high-grade ICA stenosis (> 50% according to NASCET) was very low. Analysis on the presence or absence of the spot-sign may further contribute to the phenomenon of the low secondary prophylactic effect of CEA with at least transient ocular symptoms [37, 44–46]. In an analysis of CEA specimens Howard et al. performed a semi-quantitative analysis of plaque material of high-grade ICA-stenosis of 1317 patients with cerebral stroke and 323 patients with ocular ischemia (of which 81% amaurosis fugax and 19% manifest retinal ischemia) [18]. They found patients with symptomatic high-grade stenosis and cerebral events to have significantly more features of vulnerable plaque than patients with ocular events. Unfortunately, no sub-analysis for amaurosis fugax and IR was made that would add to the analysis of our study. Detailed analysis of CRAO by ocular sonography may further help to identify the best treatment strategies and may be an ideal study population to revive comparison of modern best medical treatment versus carotid endarterectomy and carotid artery stenting.

**Fig. 6:**
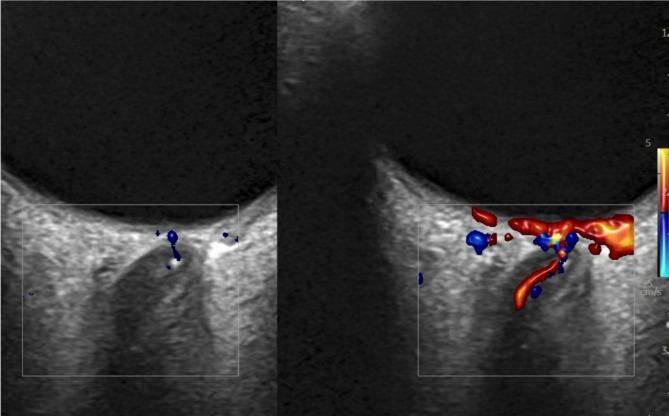
asymptomatic “spot sign” in a patient with normal flow in the central retinal artery

### Limitations

Our study suffers from limitations, mainly (a) the retrospective design with perhaps incomplete analysis of risk factors and (b) the low frequency of MRI that may underestimate the true number of concurrent stroke. For the majority of patients, no follow-up data is available. Further prospective registries should be initiated to corroborate these findings. Also, CRAO was diagnosed by the ophthalmologist on call and fundoscopic images were not always taken, making fundoscopic characterization of additional retinal emboli not possible. However, ssCRAO may even be completely asymptomatic as demonstrated by a recent patient with a completely asymptomatic 40% predominantly echogenic ICA stenosis with a spot sign and normal flow in the central retinal artery (see Fig. 6). Further controlled studies also in the normal yet elderly population is needed.

## Conclusion

Our study indicates a larger role– if not even the main role - of atherosclerosis in IR, even in early stages of atherosclerosis. The two subgroups of IR exhibit distinct atherosclerotic risk profiles, and TOAST and ESUS criteria might not be ideal to be applied on IR as they might overestimate the role of AF and underestimate the role of AS (as only stenoses > 50% are considered as relevant) in the etiology of IR. Thus, further efforts should be made to standardize the acute diagnostic work-up including OCCS and the etiological-pathological classification of CRAO in stroke units and ophthalmologic departments, that still employ the TOAST classification and often lack standardization [47, 48]. heCRAO and ssCRAO can be distinguished by OCCS, which should be regularly performed in CRAO, especially in patients undergoing thrombolysis as it may predict the therapeutic efficacy. Provided that these findings can be replicated in other cohorts, this may lead to different primary and perhaps secondary prevention strategies in patients with different stages of atherosclerosis and might be a first step towards a better understanding of the precise pathomechanism of CRAO. In addition, the ongoing randomized studies on IVT in CRAO might further help to understand the effect of thrombolytic therapies on he and ssCRAO.

## Electronic supplementary material

Below is the link to the electronic supplementary material.

Supplementary Material 1

## Data Availability

The datasets used and/or analysed during the current study are available from the corresponding author on reasonable request.

## Abbreviations

ACA: Anterior cerebral artery
AF: Atrial fibrillation
AS: Atherosclerosis
CCA: Common carotid artery
cCT: Cerebral computed tomography
CEA: Carotid endarterectomy
cMRI: Cerebral magnetic resonance imaging
CRAO: Central retinal artery occlusion
ssCRAO: Spot sign positive CRAO
heCRAO: Spot sign negative (hypoechoic) CRAO
ESUS: Embolic stroke of undetermined source
ICA: Internal carotid artery
IR: Retinal ischemia
IS: Ischemic stroke
IVT: Intravenous thrombolysis
MCA: Middle cerebral artery
NASCET: North American Symptomatic Carotid Endarterectomy Trial
OCCS: Orbital color-coded sonography
TOAST: Trial of Org 10172 in Acute Stroke
