# Supplementary material for: Retinal ischemia due to different stages of atherosclerosis - insights from a retrospective study on central retinal artery occlusion
Source: Neurol Res Pract. 2025 Jul 22;7(1):50. doi: 10.1186/s42466-025-00413-z (PMC12285047; doi:10.1186/s42466-025-00413-z)
Supplement: Supplementary file 1 — Supplementary Material 1 [file 42466_2025_413_MOESM1_ESM.docx]

**Supplement - Examples of spot-sign (ssCRAO) vs. hypoechoic central retinal artery occlusion (heCRAO) and different stages of atherosclerosis**

**Spot-sign central retinal artery occlusion with echorich atherosclerosis** Solleder, Gertud,


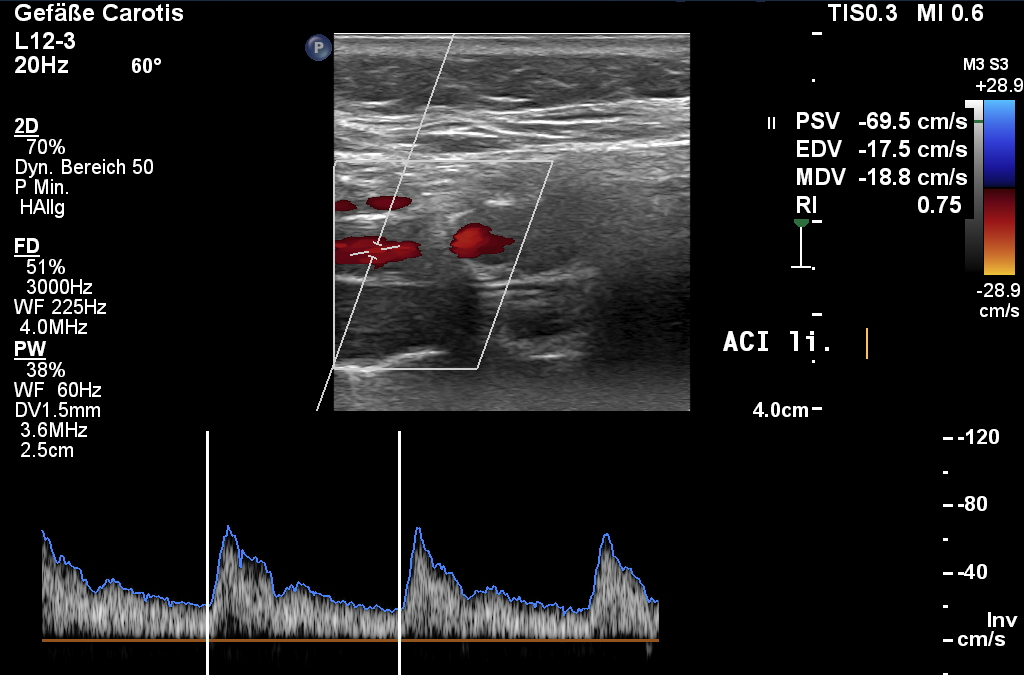

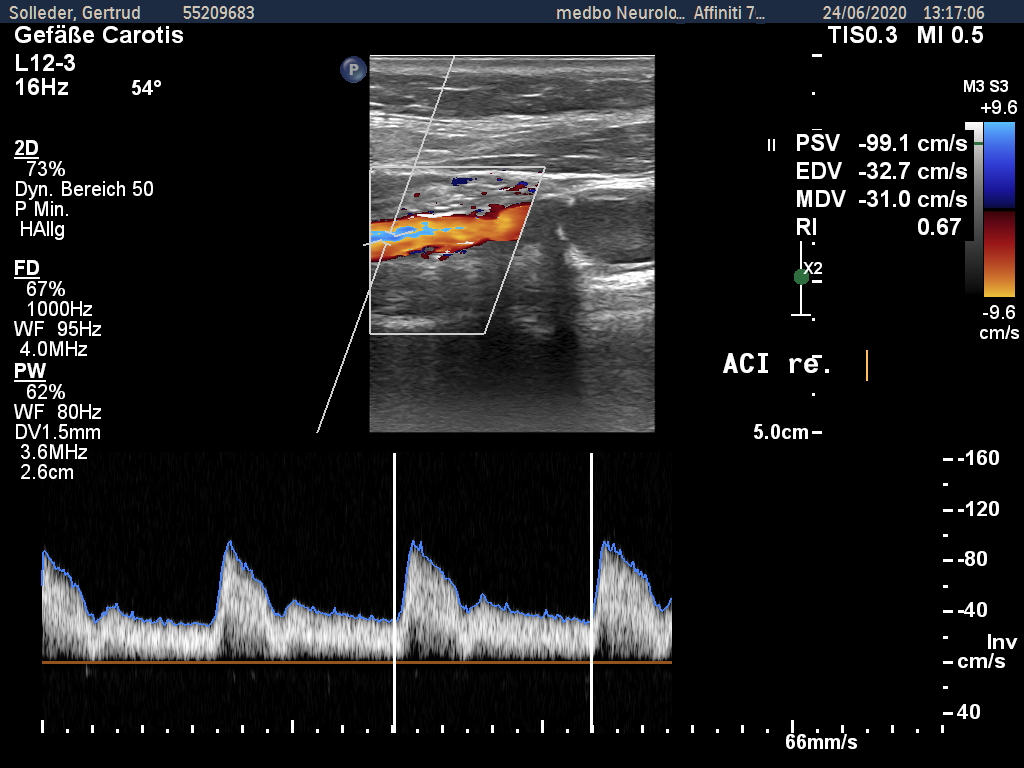

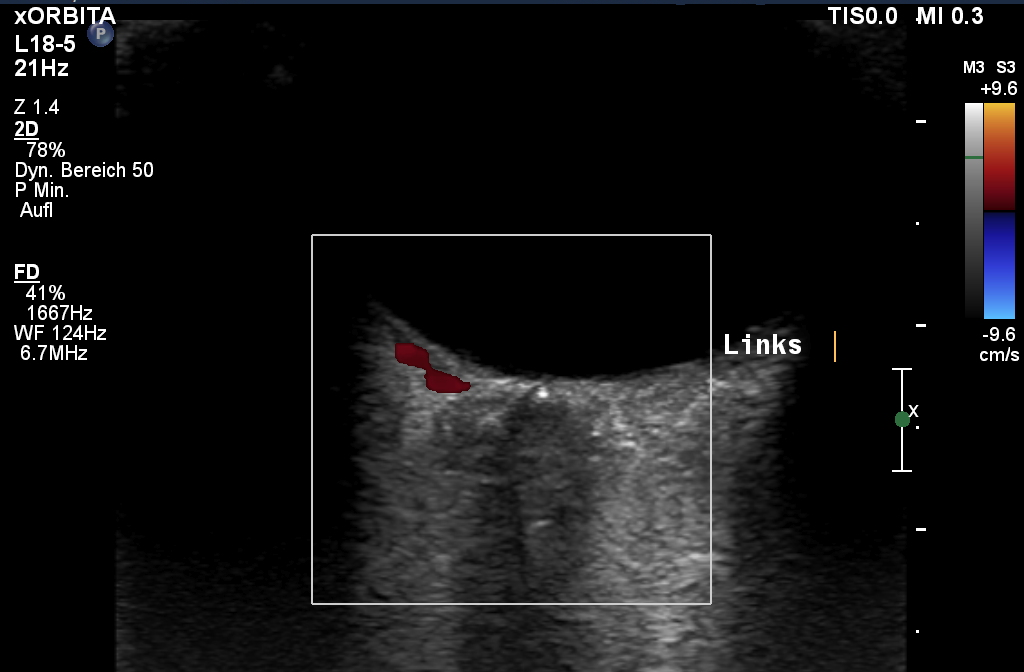


Left internal carotid artery (ipsilateral) Right internal carotid artery Spot-sign occlusion left central retinal artery

**Comment:** Both ICA have a degree of stenosis around 40% according to NASCET criteria and predominantly echorich as examplified by the shadowing artifact on B-mode sonography. On the right you see the spot-sign, absent flow in the central retinal artery but choroid arterial flow. 59yrs, female

**Hypoechoic central retinal artery occlusion with predominant echopoor atherosclerosis**


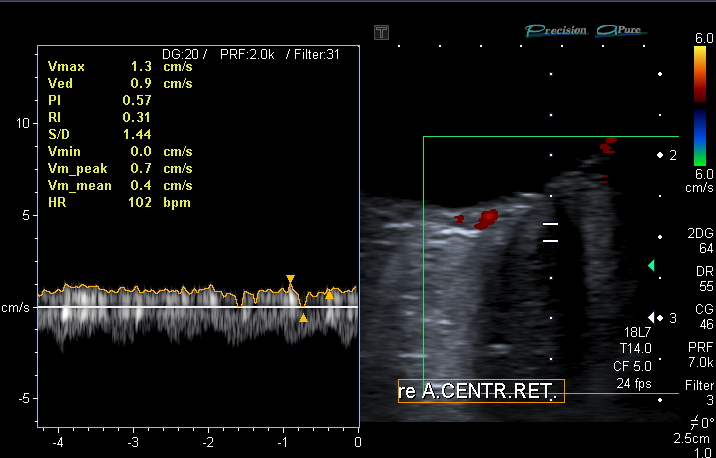

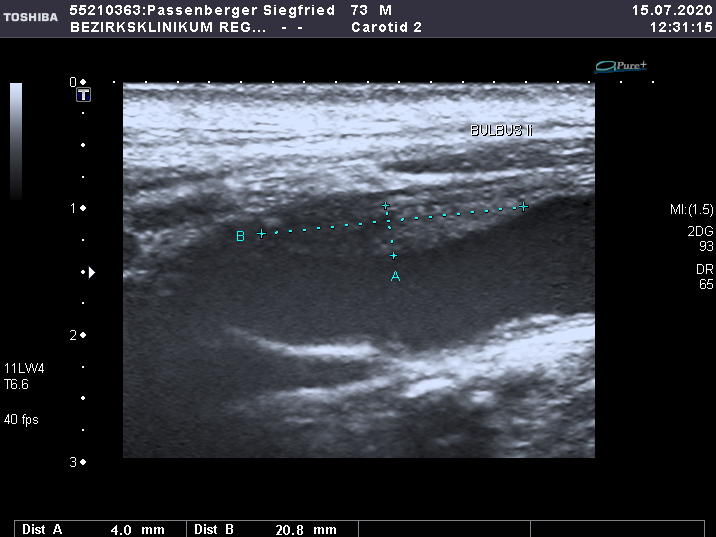

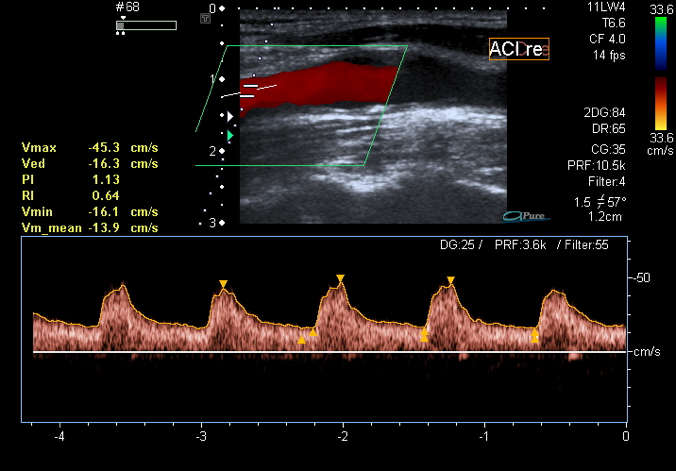


Right central retinal artery, heCRAO mainly echopoor atherosclerosis, right common and extending internal carotid artery

**Comment:** Prominent morphological 30% echopoor atherosclerosis at the ipsilateral common carotid artery while the ICA origin has a mixed long 10% plaque. 69 yrs, male patient.

**Spot-sign central retinal artery occlusion with echorich atherosclerosis with predominant echorich atherosclerosis**, **80yrs. female**


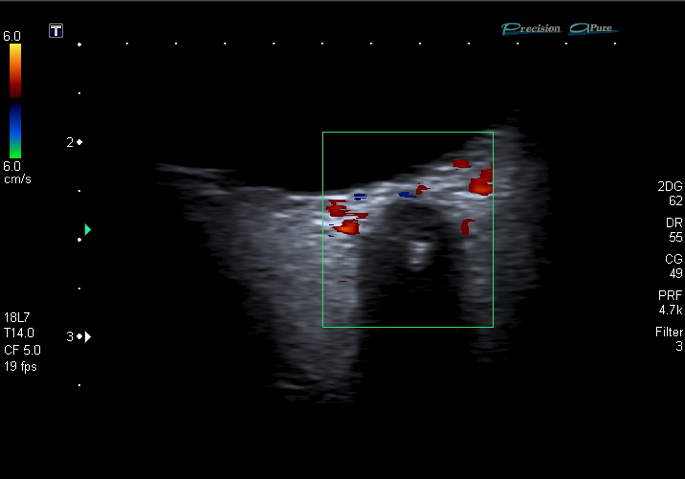

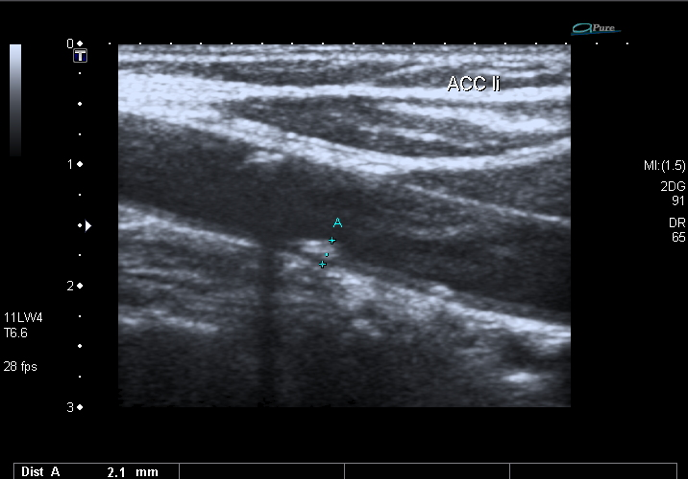

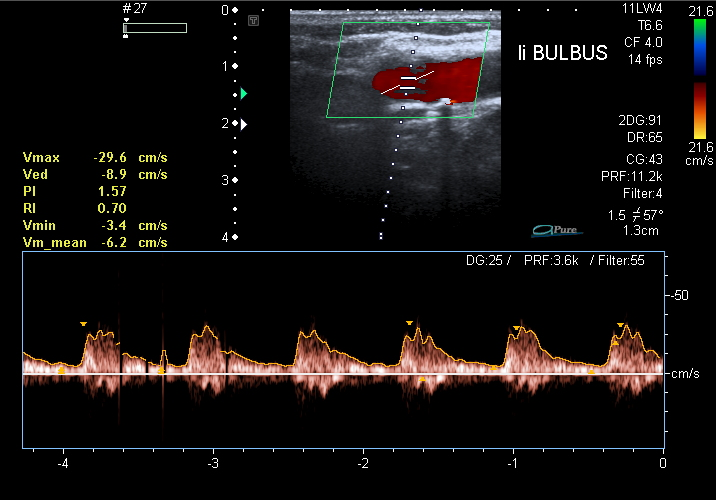


Spot-sign left central retinal artery mainly rich atherosclerosis left common and internal carotid artery (bulb)

**Hypoechoic central retinal artery occlusion with mixed low grade atherosclerosis in a 61yrs. male patient**


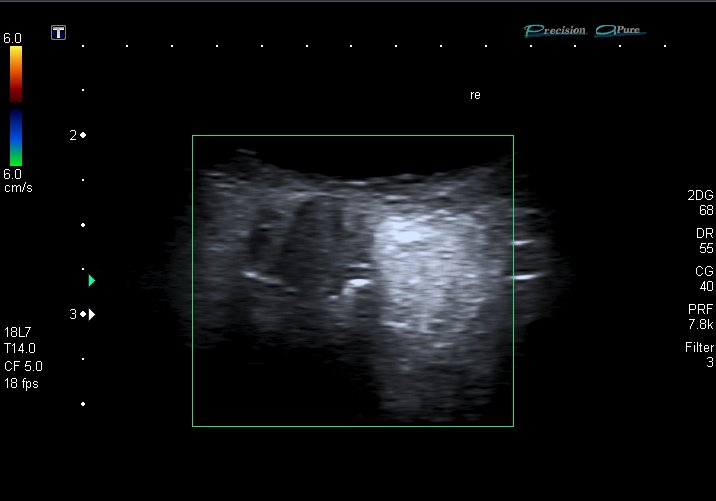

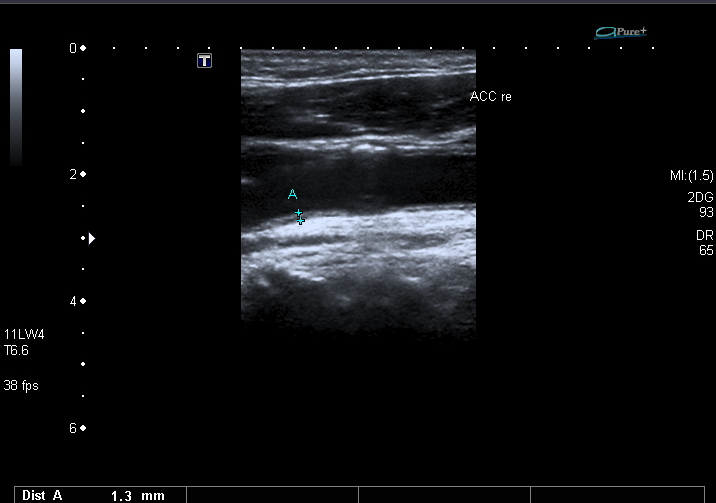

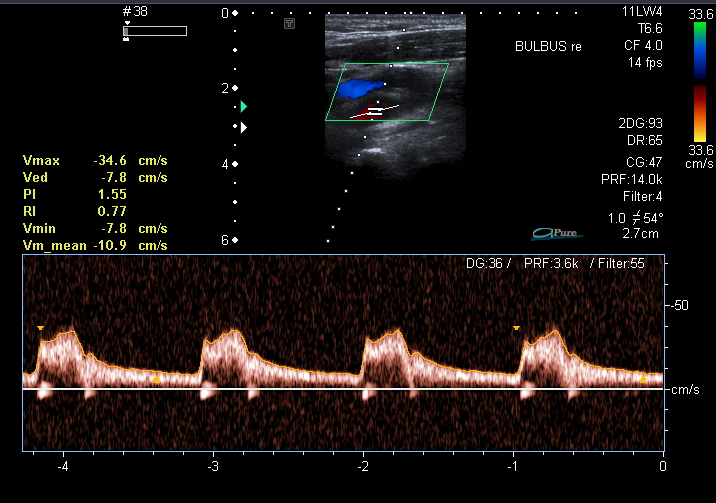


Hypoechoic central retinal artery occlusion increased intima-media thickness and small calcification, left CCA and ICA-bulb
